# Draft Genome Sequences of Biofilm-Forming and Non-Biofilm-Forming Nontyphoidal *Salmonella enterica* Serovars

**DOI:** 10.1128/genomeA.01061-17

**Published:** 2017-09-28

**Authors:** Deeksha Shetty, Alexander A. Grigoryan, Sahar Alshalchi, Niradha Withana Gamage, Julie Roy, John R. Lawrence, Sinisa Vidovic, Darren R. Korber

**Affiliations:** a Department of Food and Bioproduct Sciences, University of Saskatchewan, Saskatoon, Saskatchewan, Canada; b Department of Veterinary and Biomedical Sciences, University of Minnesota, Saint Paul, Minnesota, USA; c National Hydrology Research Centre, Environment Canada, Saskatoon, Saskatchewan, Canada

## Abstract

The genetic basis for biofilm formation among nontyphoidal salmonellae (NTS) remains poorly understood. This draft genome submission provides initial insights on the genetic differences between biofilm-forming and non-biofilm-forming clinical and environmental NTS serovars.

## GENOME ANNOUNCEMENT

Nontyphoidal salmonellae (NTS) are pathogens of global importance (1) with various virulence factors, including the ability to adhere and colonize the host’s epithelial cells as well as abiotic surfaces. Adherence, colonization, and formation of biofilms, in the host and nonhost environments, represent a key feature of the NTS life cycle that greatly influences their persistence as well as infection potential (2).

The NTS isolates sequenced in this study include pairs of strains from each of three *Salmonella enterica* serovars (Heidelberg, Typhimurium, and Dublin, obtained in Minnesota during 2015), which possess diametrically opposed biofilm phenotypes (biofilm formers and non-biofilm formers) as assessed by the crystal violet binding assay (3). The three biofilm-forming strains *S*. Heidelberg MN-714, *S*. Typhimurium variant 5 MN-74 (D15-052810), and *S*. Dublin MN-12 (D15-051537) were isolated from a poultry barn, bovine lymph node, and porcine lung, respectively. The three non-biofilm-forming strains *S*. Heidelberg MN-618, *S*. Typhimurium variant 5 MN-62 (D15-043619), and *S*. Dublin MN-69 (D15-009047) were isolated from a poultry barn, bovine brain, and porcine lung, respectively.

Genomic DNA was isolated using the DNeasy blood and tissue kit (Qiagen, USA) and processed on the MiSeq platform (Illumina, Inc., San Diego, CA, USA). Paired-end reads were *de novo* assembled by the A5-miseq pipeline (4). The assembled contigs were annotated using Rapid Annotation using Subsystem Technology (RAST) server version 2.0 (5) (http://rast.nmpdr.org), and PGAP, of the NCBI Prokaryotic Genome Annotation Pipeline (http://www.ncbi.nlm.nih.gov/genomes/static/Pipeline.html). The occurrence of antibiotic resistance genes, clustered regularly interspaced short palindromic repeat (CRISPR) regions, prophages, *Salmonella* pathogenicity islands (SPIs), and number of plasmids on assembled sequences were predicted using ResFinder version 2.1 (6), CRISPRFinder (http://crispr.i2bc.paris-saclay.fr/Server/), PHAST (7), SPI Finder version 1.0 (https://cge.cbs.dtu.dk/services/SPIFinder), and PlasmidFinder version 1.3 (8), respectively (Table 1).

**TABLE 1:** Statistical information for draft genome sequences

Organism	Total size (bp)	No. of contigs	Biofilm (+/−)	Coverage (×)	No. of CDSs, rRNAs, and tRNAs	Antibiotic resistance gene(s)	No. of prophages	No. of CRISPR systems	SPI(s)	Plasmid(s)	Accession no.
*S.* Heidelberg MN-714	4,879,603	61	+	196	4,919; 10, 12, 18 (5S, 16S, 23S); 82	Beta-lactam, *blaCMY-2*	5 (2 regions intact, 2 regions incomplete, 1 region questionable)	3	SPI 13, SPI 14	*ColpVC*, *IncI1*, *IncX1*	NOXM00000000
*S*. Heidelberg MN-618	4,771,233	51	−	142	4,785; 10, 11, 19 (5S, 16S, 23S); 81	None	4 (2 regions intact, 1 region incomplete, 1 region questionable)	3	SPI 13, SPI 14	*ColpVC*, *IncX1*	NOXL00000000
*S*. Typhimurium variant 5 MN-74 (D15-052810)	5,007,586	120	+	57	5,173; 9, 10, 13 (5S, 16S, 23S); 81	Aminoglycoside, *strA*, *strB*, *aph(3′)-la*, *aadB*; beta-lactam, *blaTEM-1B*; phenicol, *cmlA1, floR*; sulfonamide, *sul2*; tetracycline, *tetA*	9 (4 regions intact, 5 regions incomplete)	2	SPI 14, SPI 5, SPI 13	*IncA/C2*, *IncFII(S)*, *IncFIB(S)*	NOXI00000000
*S*. Typhimurium variant 5 MN-62 (D15-043619)	4,934,925	66	−	96	5,022; 8, 12, 18 (5S, 16S, 23S); 82	Aminoglycoside, *aadA*, *strA*, *strB*; sulfonamide, *sul1*; tetracycline, *tetA*	12 (5 regions intact, 6 regions incomplete, 1 region questionable)	3	SPI 14, SPI 13 (LysR family transcriptional regulation; *gtrB*), SPI 13 (transcriptional regulation; *gtrA*)	None	NOXH00000000
*S*. Dublin MN-D12 (D15-051537)	4,996,886	59	+	125	4,854; 9, 6, 12 (5S, 16S, 23S); 76	Aminoglycoside, *strA*, *strB*; beta-lactam, *blaTEM-1B*, *blaCMY-2*; phenicol, *floR*; sulfonamide, *sul2*; tetracycline, *tetA*	7 (4 regions intact, 3 regions incomplete)	1	SPI 13	*IncA/C*, *IncFII(S)*, *IncX1*	NOXK00000000
*S*. Dublin MN-D69 (D15-009047)	4,767,053	66	−	67	4,833; 9, 15, 9 (5S, 16S, 23S); 80	Aminoglycoside, *aadA2*, *aph(3′)-la*, *strA*, *strB*; beta-lactam, *blaCMY-2*; macrolide, lincosamide, and streptogramin B, *mph(A)*; phenicol, *floR*; sulfonamide, *sul1*, *sul2*; trimethoprim, *dfrA12*	6 (4 regions intact, 2 regions incomplete)	2	None	*IncA/C2*	NOXJ00000000

Our data reveal that biofilm-forming NTS strains have larger genome sizes and increased coding sequences (CDSs) relative to non-biofilm formers. A larger genome size would favor increased functional gene content and could enhance the ability of organisms to form biofilms under stressful conditions. It is also noteworthy that the biofilm-forming strains *S*. Heidelberg MN-714 (*ColpVC, IncI1*, and *IncX1*), *S*. Typhimurium variant 5 MN-74 (D15-052810) [*IncA/C2*, *IncFII(S)*, and *IncFIB(S)*], and *S*. Dublin MN-D12 (D15-051537) [*IncA/C*, *IncFII(S)*, and *IncX1*] possessed a higher number of plasmids than the non-biofilm-forming strains *S*. Heidelberg MN-618 (*ColpVC* and *IncX1*), *S*. Typhimurium variant 5 MN-62 (D15-043619) (no plasmid), and *S*. Dublin D MN-69 (D15-009047) (*IncA/C2*). Biofilms provide an ideal niche for plasmid exchange and also promotes plasmid stability (9). Furthermore, plasmids induce biofilm formation by encoding factors required for biofilm formation (10).

These findings suggest that detailed analysis of genetic diversity and variable gene expression in biofilm-forming and non-biofilm-forming NTS strains could aid in identifying serovar-specific genes/plasmids, offering the potential for elucidating the molecular basis for biofilm formation and targeted biofilm control.

### Accession number(s).

Whole-genome sequences of the six NTS isolates *S*. Heidelberg MN-714, *S*. Typhimurium variant 5 MN-74 (D15-052810), *S*. Dublin MN-12 (D15-051537), *S*. Heidelberg MN-618, *S*. Typhimurium variant 5 MN-62 (D15-043619), and *S*. Dublin MN-69 (D15-009047) have been deposited in DDBJ/ENA/GenBank under the accession numbers listed in Table 1. The versions reported here are the first versions.
